# Sugar of Milk

**Published:** 1891-08-08

**Authors:** 


### The Practitioner's Mirror and Retrospect.

[*The Editor will be glad to receive offers of co-operation and contributions from members of the profession. All letters should be addressed to* The Editor, The Lodge, Porchester Square, London, W.]

### MEDICINE.

### SUGAR OF MILK AS A DIURETIC.

Some of our best diuretics seem to have been discovered in unexpected quarters. We found the gypsies using digitalis and probably broom-tops long before they were known to have specific properties. The Russian peasant employed the cockroach, the American Indian the chimaphila. According to some authorities the squill was numbered among the divinities of the Egyptians, and the potash salts were certainly first prescribed in the ashes of various sacrifices. The therapeutic virtues of milk or whey have of late been forgotten in its dietetic value, and the existence of sugar of milk in the pharmacopœias has the air of an interesting survival, justified by its harmless character and the inert powder it affords for the administration of active drugs. The wheys and possets of another age have been so forgotten, that their supposed virtues are only made out by antiquarian research, but all at once we find sugar of milk reinstated by a great French physician, Germain Sée, as one of the most powerful diuretics which we possess. If his results are borne out by the purgatorial period of practical testing in which so many new drugs disappear, we shall have especial cause for rejoicing. For the substance, it is at once cheap and easily procured, harmless, and without the horrible taste or odour which detract from the value of so many other remedies, less soluble than cane sugar, and not directly subject to alcoholic fermentation, it forms a peculiar gritty mass crystallising in four-sided prisms. Practically it is chiefly brought from Switzerland, where the whey is concentrated and left to crystallise out in a bright yellow mass, which is afterwards decolourised by animal charcoal and repeated crystallisations. In this country it is usually seen as a mass of dirty white crystals surrounding a string on which they have been deposited. Buttermilk has been proposed as a diet in diabetes, and another milk product, the Koumis of the Russian steppes, has a certain well-recognised value in several wasting diseases. In the use of an exclusive milk diet where large quantities are consumed it had been already noticed that sometimes a considerable diuresis set in, but this was sometimes accompanied by the appearance of sugar in the urine when the diet was maintained for a long time. Naturally its use in diabetes proved anything but satisfactory, and has been almost universally abandoned. Germain discovered that the diuretic effect was produced almost entirely by the action of the sugar, and that even the salts contained in it have a relatively unimportant effect. Moreover, the lactose can be administered alone without producing the digestive disturbances with the glycosuria and azoturia which form such a serious drawback to the use of large quantities of milk. When he gave three or four ounces of milk sugar in place of the two litres of milk which some of his patients had been taking, he found daily a secretion of two, three, and even more than four litres of urine without the troubles which we have mentioned. He therefore prescribes, in certain cases, 100 grammes of milk sugar, or about three ounces daily, and says that the œdema and ascites in proper cases vanish completely. It is chiefly in diseases of the heart that these results are obtained where the kidney affection is slight or non-existent. On the other hand, in dropsy following Bright's disease there is little, if any, remedial effect to be noticed. It would appear, therefore, to be one of those which require undamaged kidney cells by which to produce diuresis. He even proposes it as a test of the extent to which disease of the kidneys is present as a concomitant of the cardiac affection. There is generally little difficulty in getting patients to take it, for the sweetness, which is almost the only taste it possesses, is not so powerful as that of cane sugar, and can be easily made more palatable by the addition of peppermint, or spirit with some aromatic. Indeed, it may be made up in the shape of puddings, or administered in slightly acidulated water. See gives the three and a-half ounces which he advises in four pints of water, and cuts off the supply of other drinkables. The diuresis is greater than that produced by any known remedy. Every practitioner will welcome the addition of a new agent to supplement the ordinary digitalis and bitartrate of potash, and to put off or replace the troublesome process of aspiration and drainage, with their accompanying risks. Even when the ordinary drugs do not lose their power, a change becomes necessary after a time from their frequently irritating effect on the digestion. Sée administers the lactose for a period of eight or ten days, and after stopping it for perhaps a week resorts again to its use. Meanwhile, we may employ the old compound digitalis pill, and similar remedies, with greater success as the patient's general health has mended with his digestion.

Dr. Meilach has made further experiments on its use and finds some result, though a limited one, even where the urine is albuminous. The extraordinary diuretic action is not confined to lactose alone, but is also found in glucose, though probably in a less degree.

Sée pointed out that the action is produced with a rise of the blood pressure by a direct influence on the secreting elements of the kidney, as he considers to be the case in theobromin, caffein, and similar bodies. It differs from this class of drugs, however, in its not producing any disturbances of the nervous system, while it is more powerful than even digitalis and convallaria. The most commonly helped cases are those of failure of compensation in heart disease, though the action of the heart indeed is in no degree improved. Sée was particularly delighted with the results of administering it combined with a little iodide of potash when severe dyspnœa accompanied the dropsy, as his cases were doubly relieved with extraordinary success. The only troublesome symptom which has been hitherto noticed is the occurrence of occasional diarrhœa and sweating, but of course these aid rather than interfere with the desired action. Buttermilk is probably only another form of administering lactose, and where patients do not object to the taste good results can be obtained. Probably, however, we are safer in giving lactose alone, as considerable quantities must be injected to produce the desired effects. Recent observations show that there is still uncertainty as to the dosage of milk sugar itself. We have ourselves seen some good results in the last six months from its employment with Sée's dose of 3½ ounces daily in cardiac cases, while in other instances there seemed little amendment. The contentment with which patients take it, and the absence of any troublesome disturbances, certainly appeared one of the great advantages of the drug. Zuvadsky considers the dose is too high, and uses only 3 to 4½ drachms daily. However, careful investigation is needed, and we imagine that within a short time full and complete observations will be made both as to the dosage and the physiological action of both lactose and glucose. The much-vaunted grape cure would seem to be only another instance of the employment of glucose, though the mode of life, the purely vegetable diet, have a share in the result. We have been able to note little as yet of the value of lactose in cases of liver dropsy, but, so far as we have observed, there appeared to be some reduction of the fluid. In purely heart cases of certain types there is, we think, good evidence of this last addition to our pharmacopœia.

